# Supplementary figures and images for: Comprehensive analysis of lectin-glycan interactions reveals determinants of lectin specificity
Source: PLoS Comput Biol. 2021 Oct 6;17(10):e1009470. doi: 10.1371/journal.pcbi.1009470 (PMC8523061; doi:10.1371/journal.pcbi.1009470)

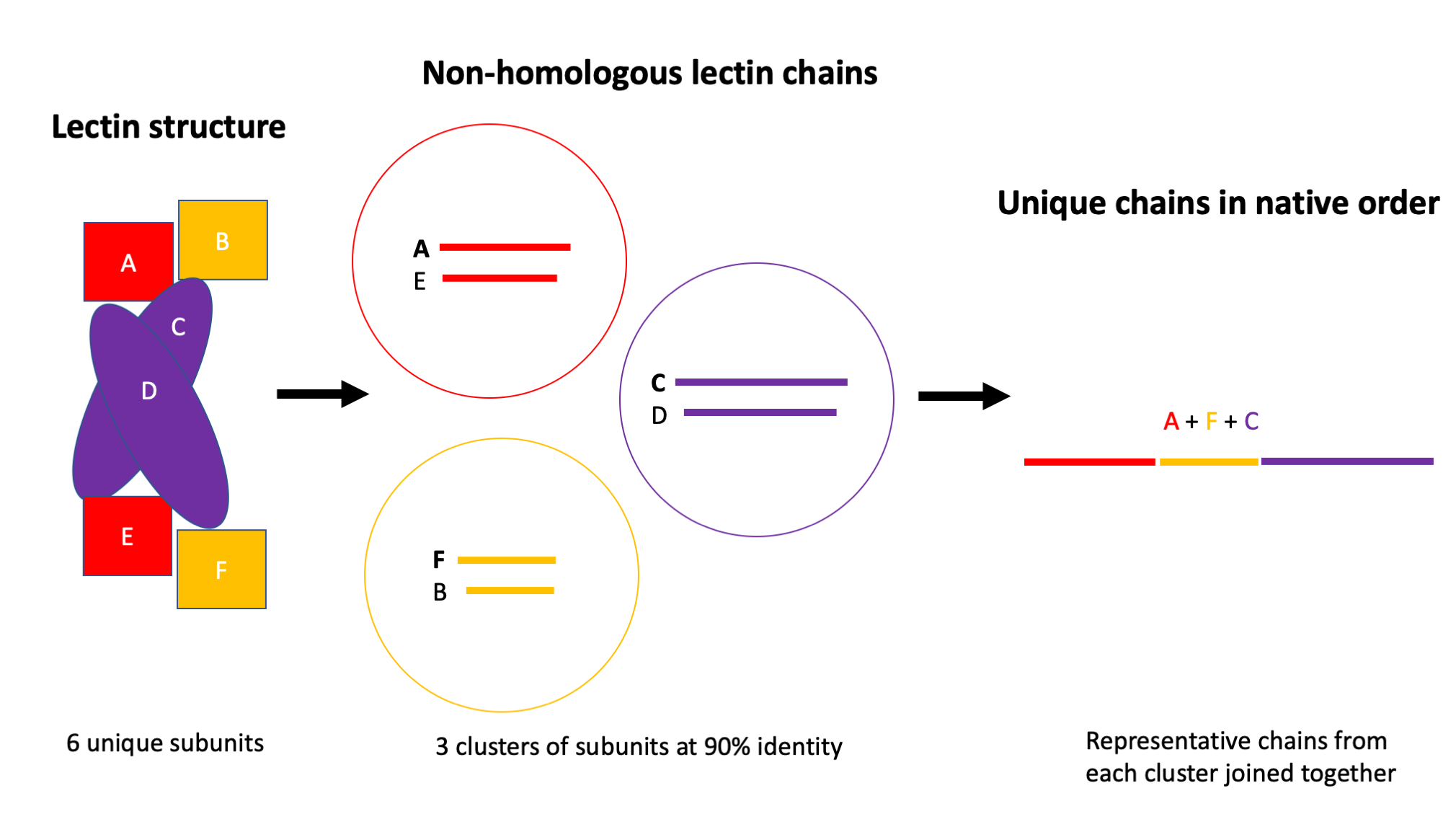

Supplement: S1 Fig — Schematic of the workflow used to extract non-redundant sequences from structure files. (TIF) [file pcbi.1009470.s002.tif]

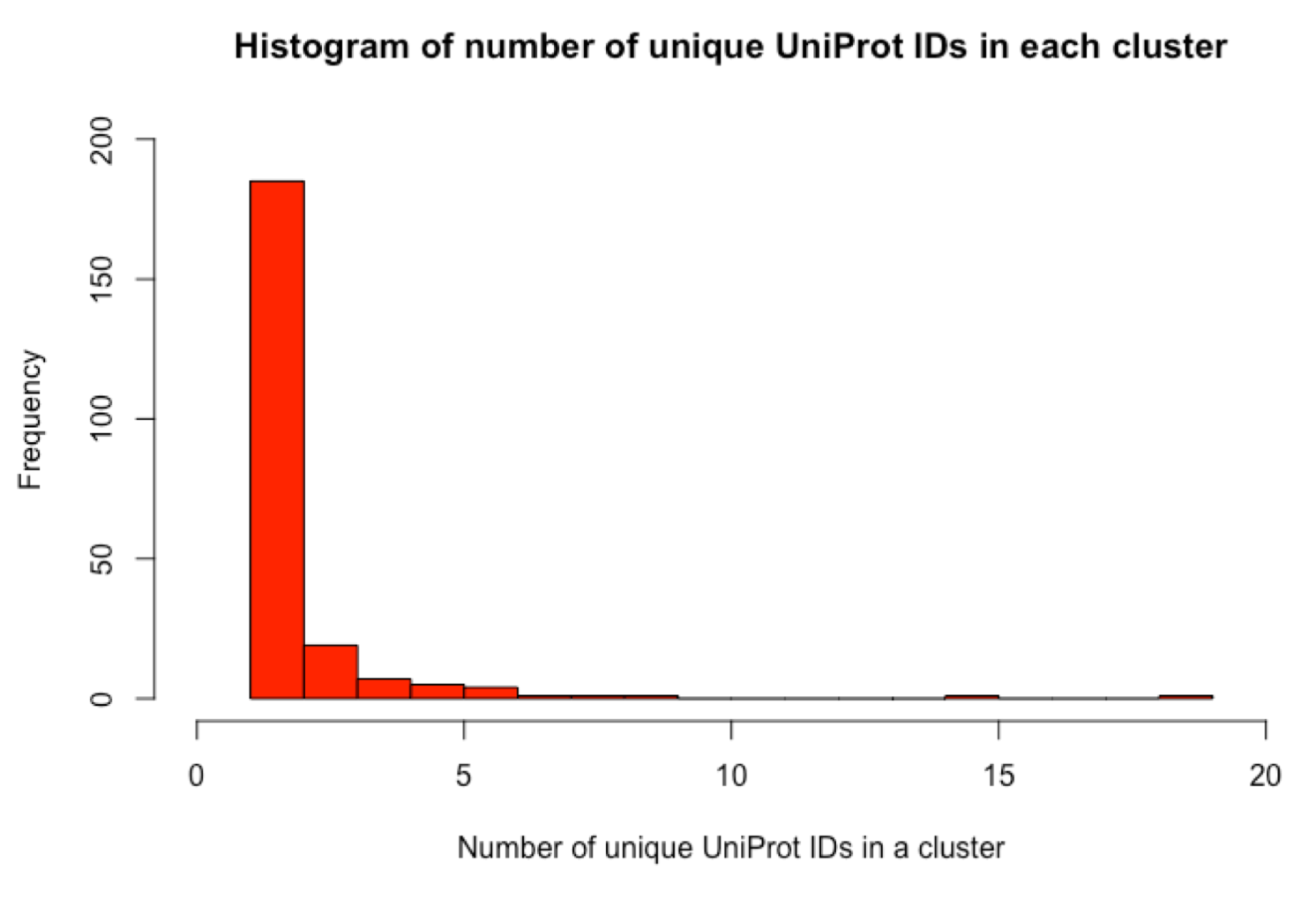

Supplement: S2 Fig — The number of unique lectins (as defined by UniProtID) within each homology cluster generated with CD-HIT at 50% sequence identity. Most homology clusters only contained 5 or fewer unique lectins, but some very well studied lectins and homologous lectins were grouped into very large homology clusters. (TIF) [file pcbi.1009470.s003.tif]

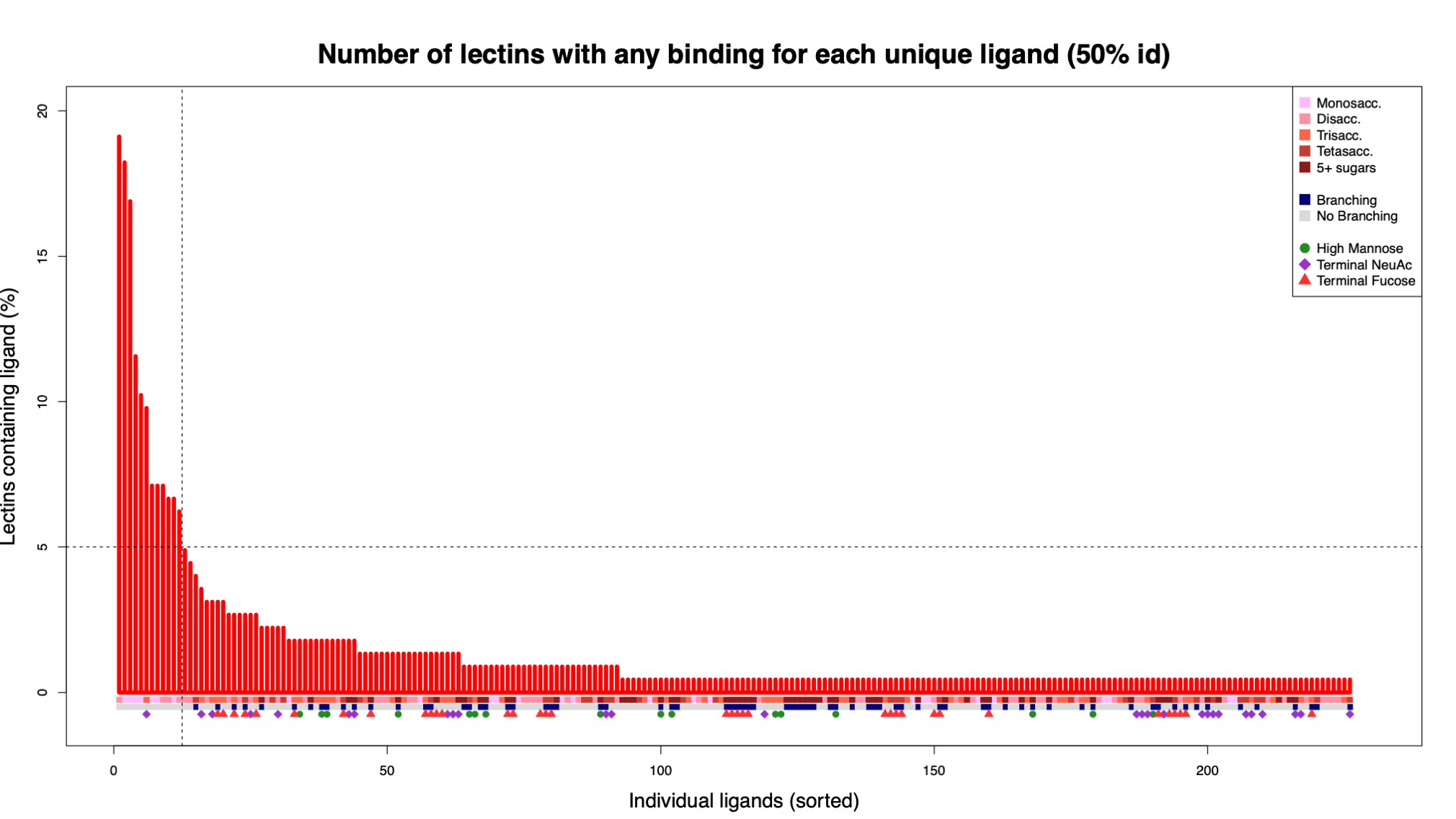

Supplement: S3 Fig — Frequencies of all 226 unique IUPAC-labelled glycans within each cluster of homologous lectins. The top 12 individual glycans (vertical line) each appeared in complex with at least 5% of the 225 clusters of homologous lectins (horizontal line). Information about each glycan is provided below each bar of the barplot, including membership of one of the three groups of glycans (terminal NeuAc, high mannose, and terminal fucose). (TIF) [file pcbi.1009470.s004.tif]

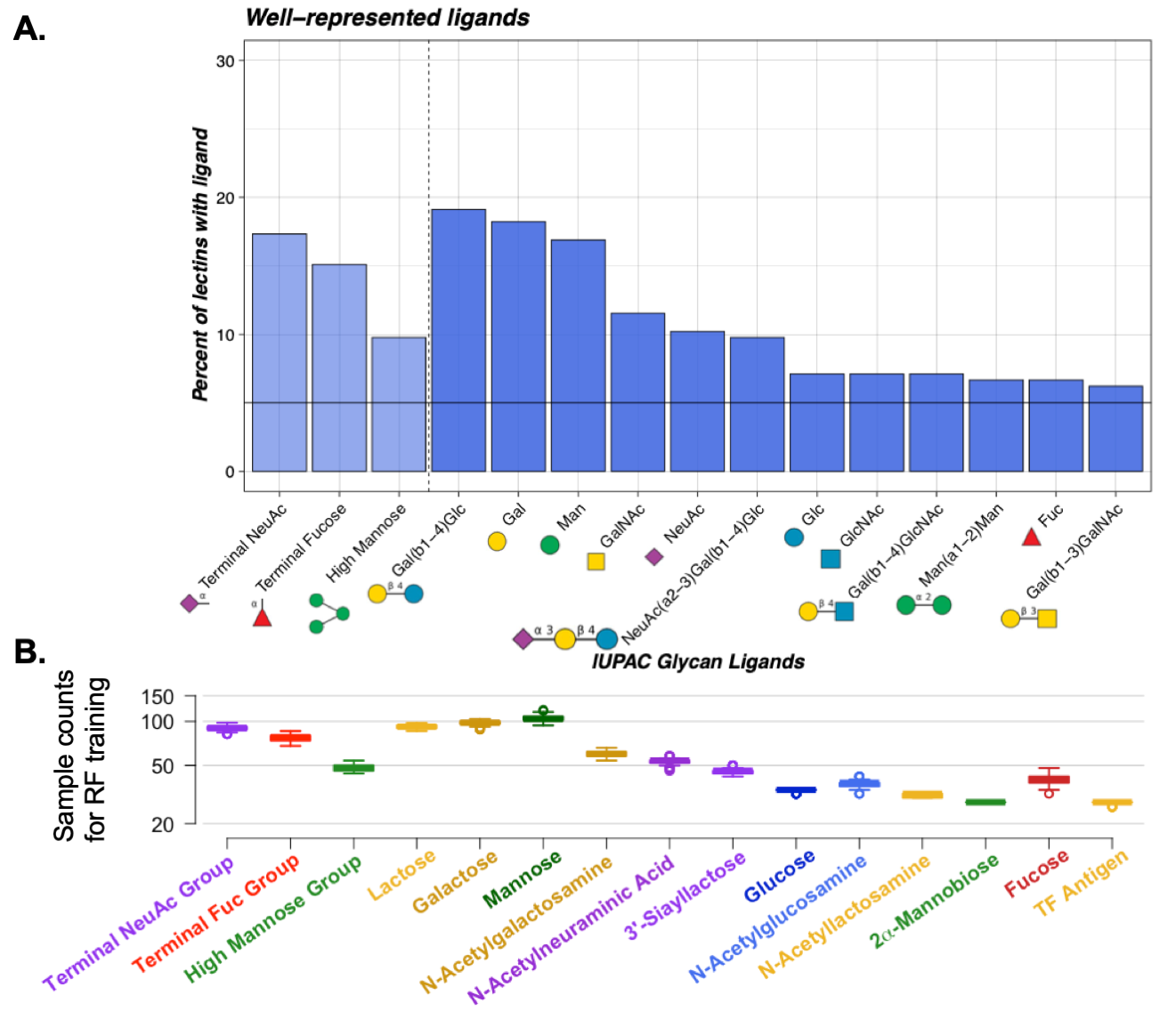

Supplement: S4 Fig — Panel A shows the same values as S3 Fig for the 12 most commonly bound glycans (right of the dotted vertical line), annotated with their corresponding IUPAC names and SNFG symbols, as well as the frequencies of the 3 groups of glycans (left of the dotted vertical line) appearing bound to any lectins in the 225 homology clusters with their representative SNFG symbols. Panel B shows the actual distributions of training samples (on a log scale) used for each individual RF model for each glycan at reach repeat and interaction of the leave-one-out cross-validation. These distributions appeared fairly proportional to the relative frequencies of each glycan in panel A. (TIF) [file pcbi.1009470.s005.tif]

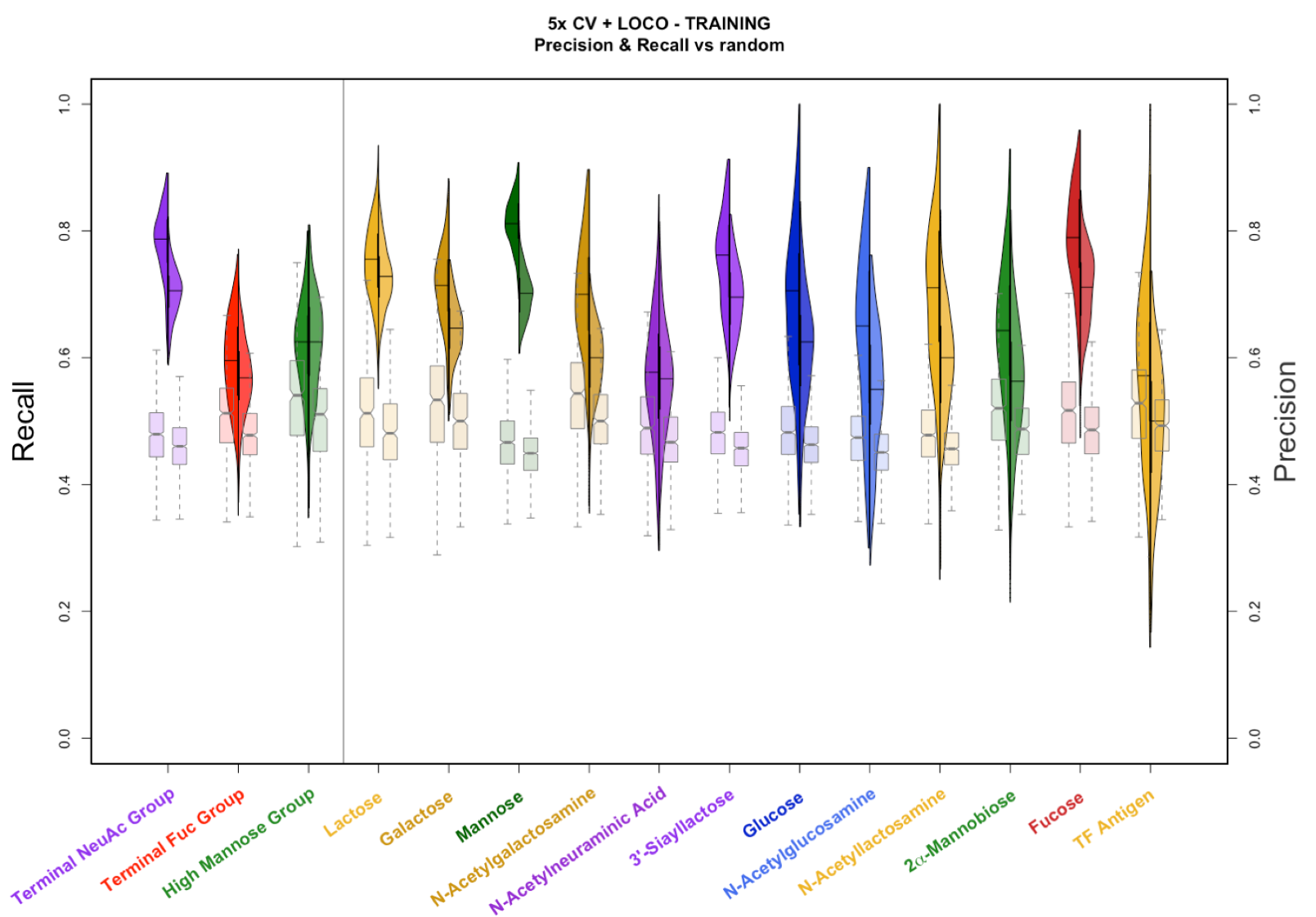

Supplement: S5 Fig — Training performance of glycan-specific RF models measured with nested 5x cross-validation. Recall (left y-axis) and precision (right y-axis) of glycan-specific random forest models is shown by the split violin plots, with the left-hand distributions depicting recall and the right-hand distributions depicting precision. The pairs of notch boxplots for each glycan show the performance of the random classifiers trained on data with shuffled labels, where again the left-hand boxplots depict the random classifiers’ recall and the right-hand boxplots depict their precision. (TIF) [file pcbi.1009470.s006.tif]

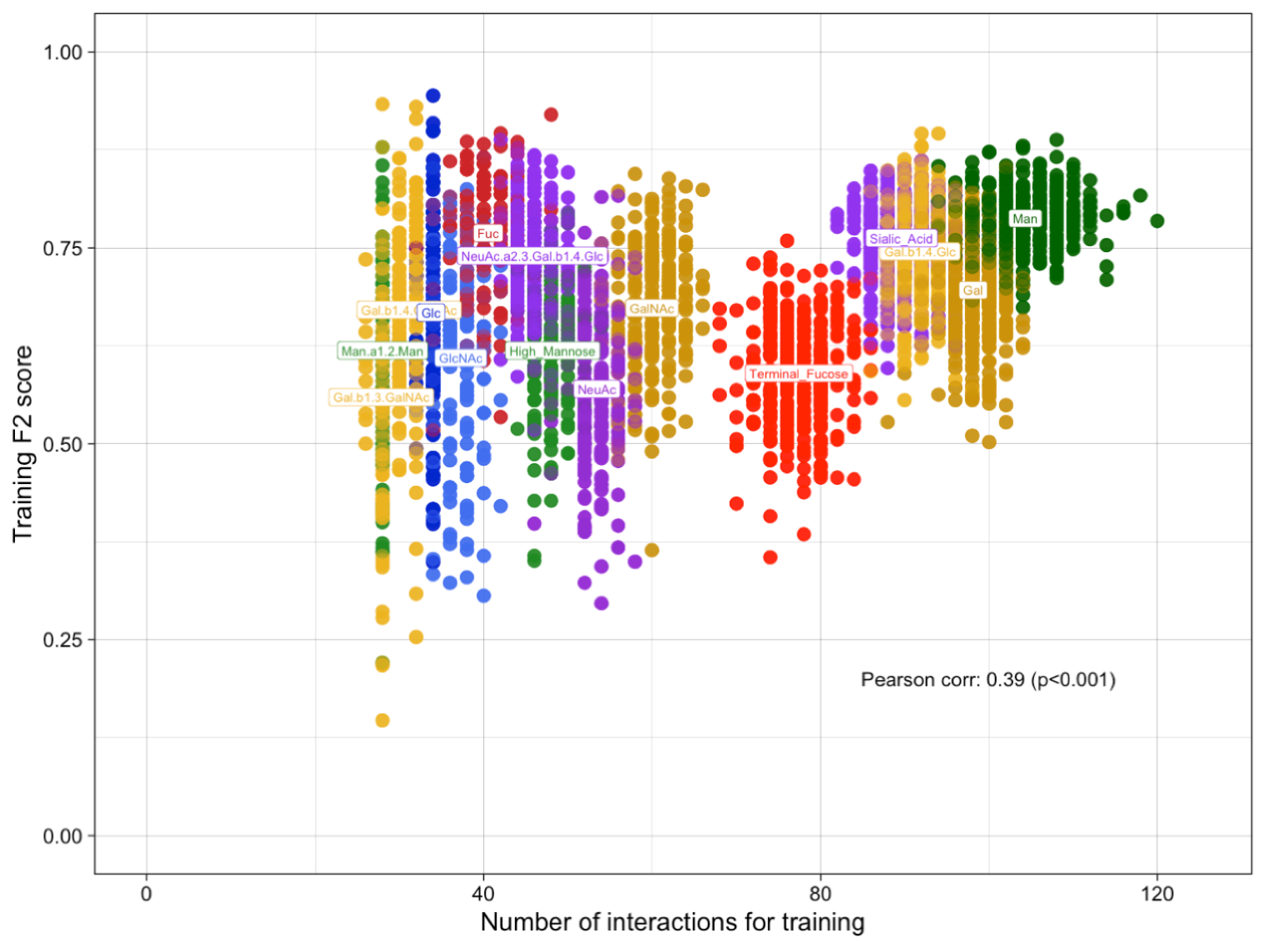

Supplement: S6 Fig — Training performance of glycan-specific RF models summarized by F2 scores combining recall and precision with greater emphasis on recall, plotted against the number of samples used in training each specific model. Glycan labels are placed on the mean F2 and sample numbers for each glycan. Training nested-cross-validation performance is fairly correlated with the number of samples available for training (Pearson correlation ρ = 0.39, p < 0.001). (TIF) [file pcbi.1009470.s007.tif]

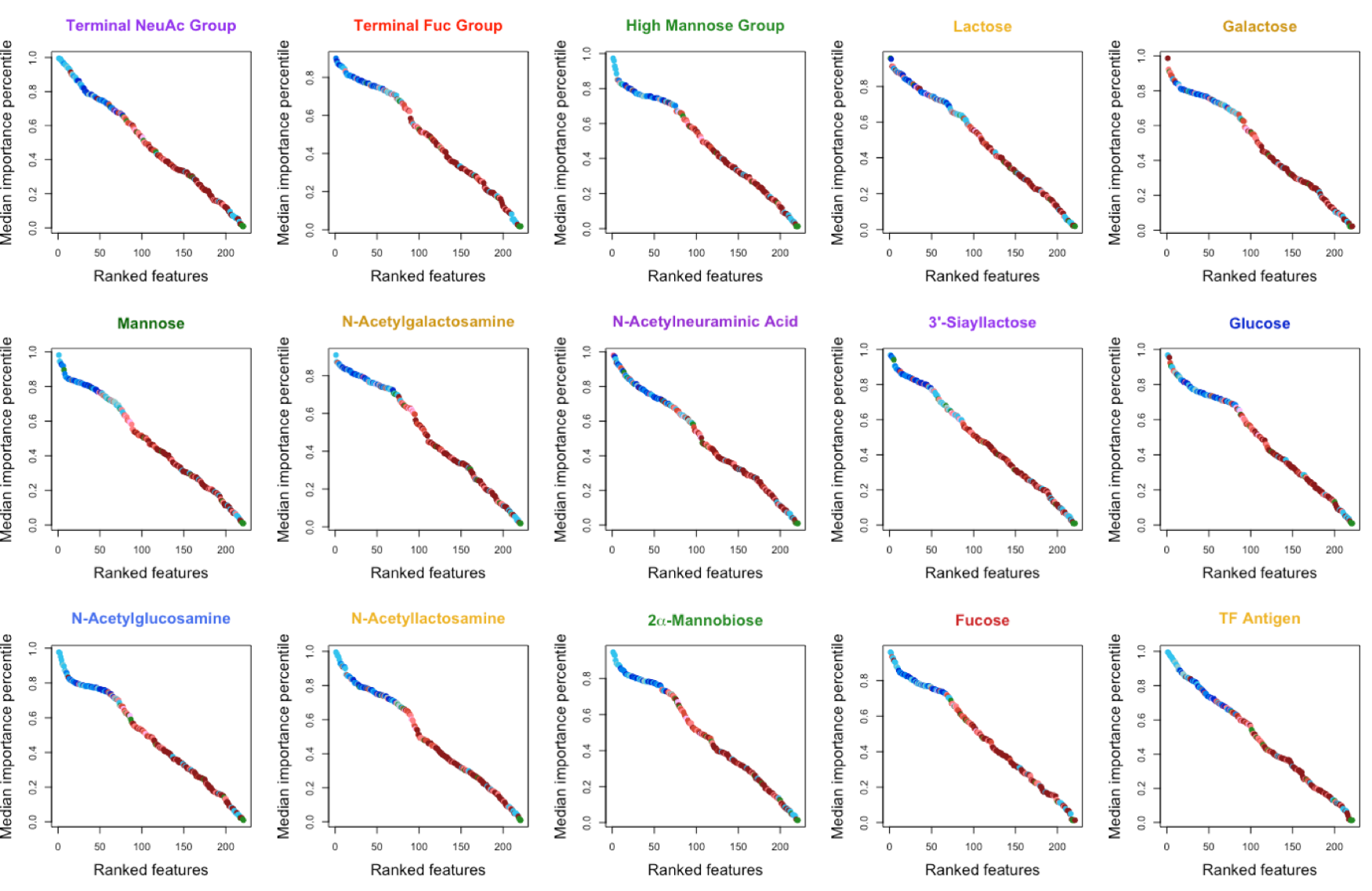

Supplement: S7 Fig — Median feature importance percentiles from each glycan-specific RF model determined via mean decrease in Gini impurity. Size-correlated pocket features (blue) were often grouped together at a higher importance level, motivating the stratification by feature type to prevent multicollinearity from one feature type affecting other features. Points were colored with the same color scheme detailed in Figs 1, 2, 4 and 6. (TIF) [file pcbi.1009470.s008.tif]

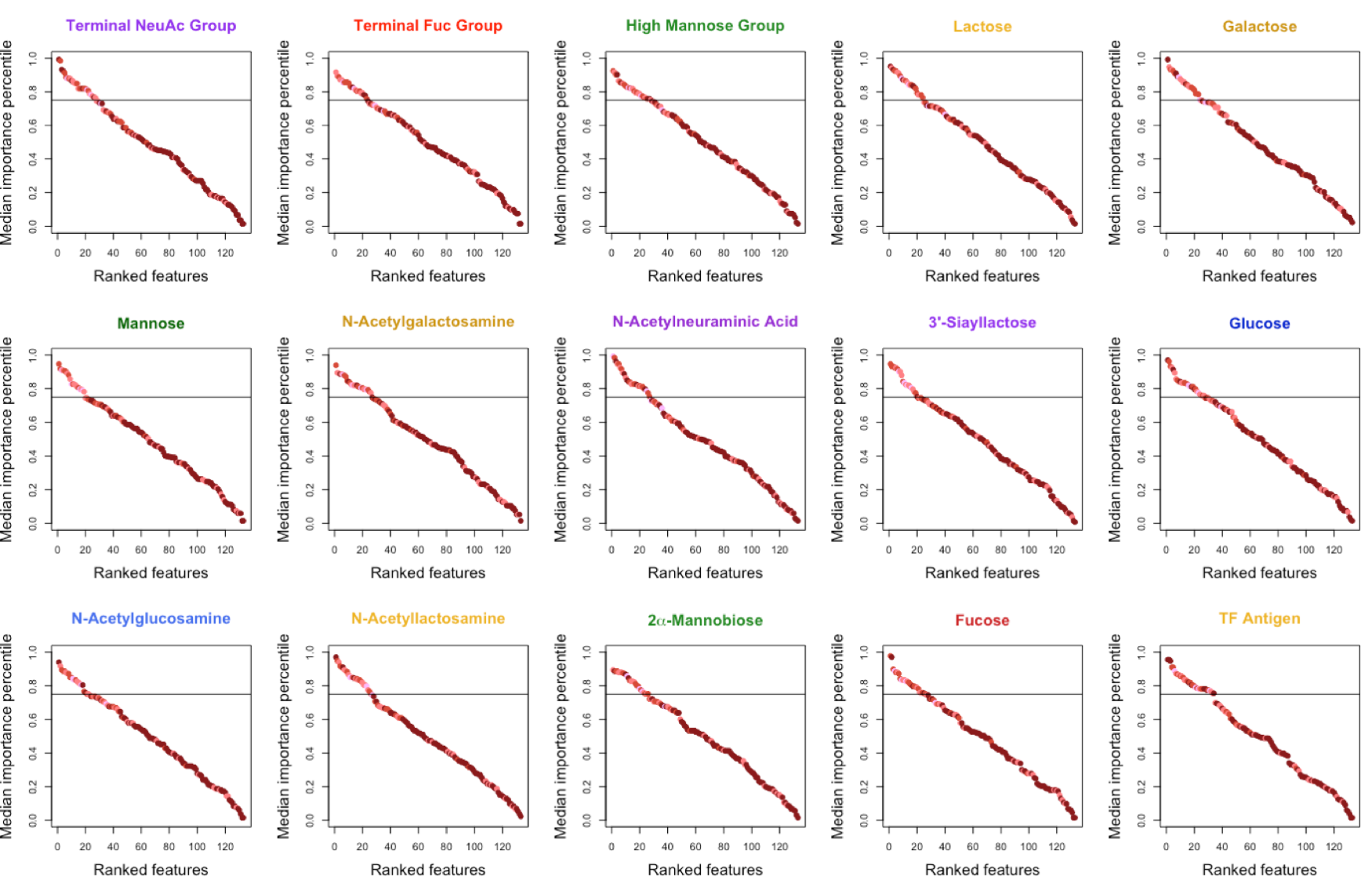

Supplement: S8 Fig — Median feature importance percentiles of residue-based features (within the residue features only) from each glycan-specific RF model determined via mean decrease in Gini impurity. Features with median importance in at least the 75th percentile (horizontal line) were considered highly predictive. Points were colored with the same color scheme detailed in Figs 1, 2, 4 and 6. (TIF) [file pcbi.1009470.s009.tif]

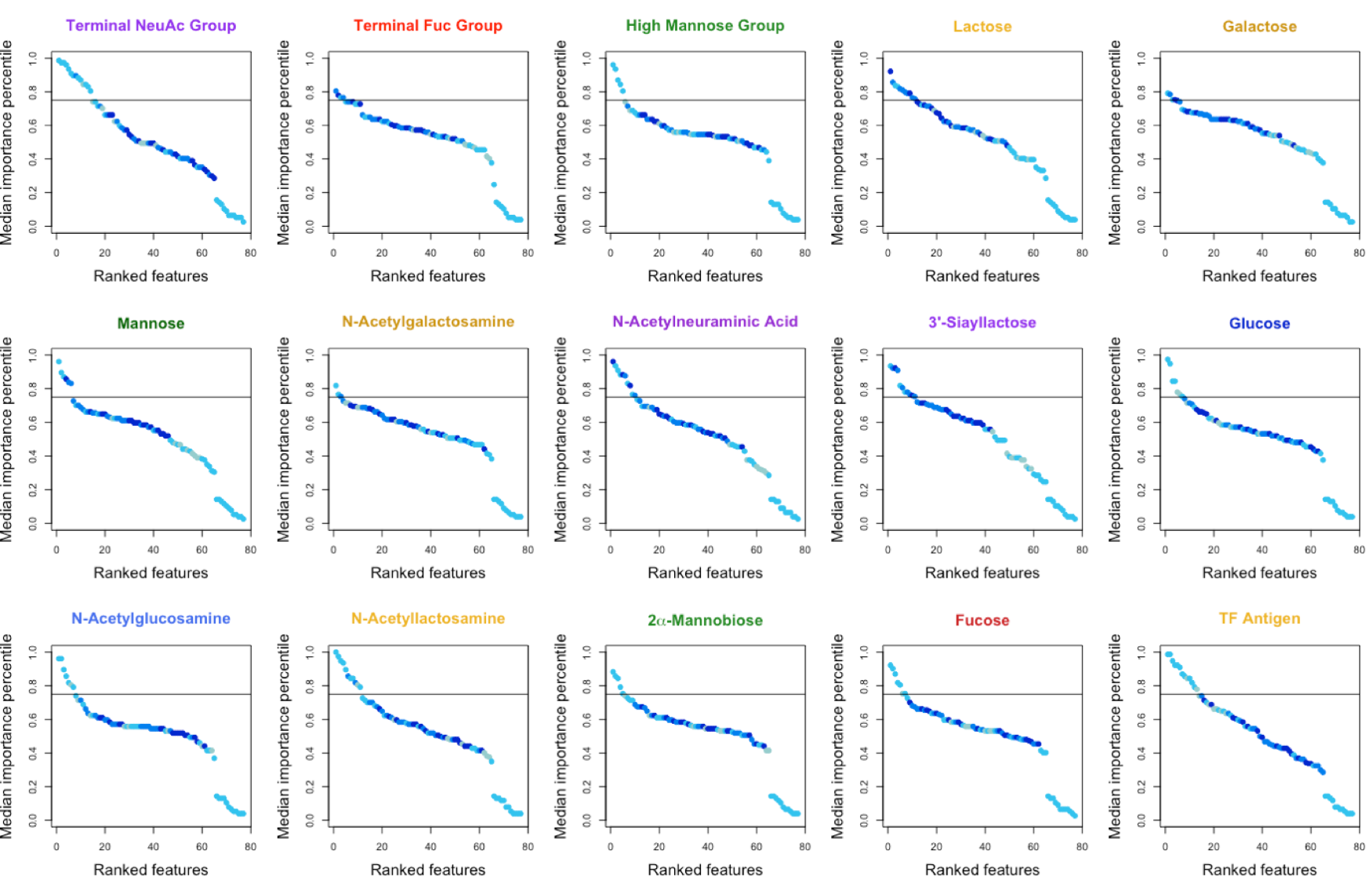

Supplement: S9 Fig — Median feature importance percentiles of pocket-based features (within the pocket features only) from each glycan-specific RF model determined via mean decrease in Gini impurity. Features with median importance in at least the 75th percentile (horizontal line) were considered highly predictive. Points were colored with the same color scheme detailed in Figs 1, 2, 4 and 6. (TIF) [file pcbi.1009470.s010.tif]

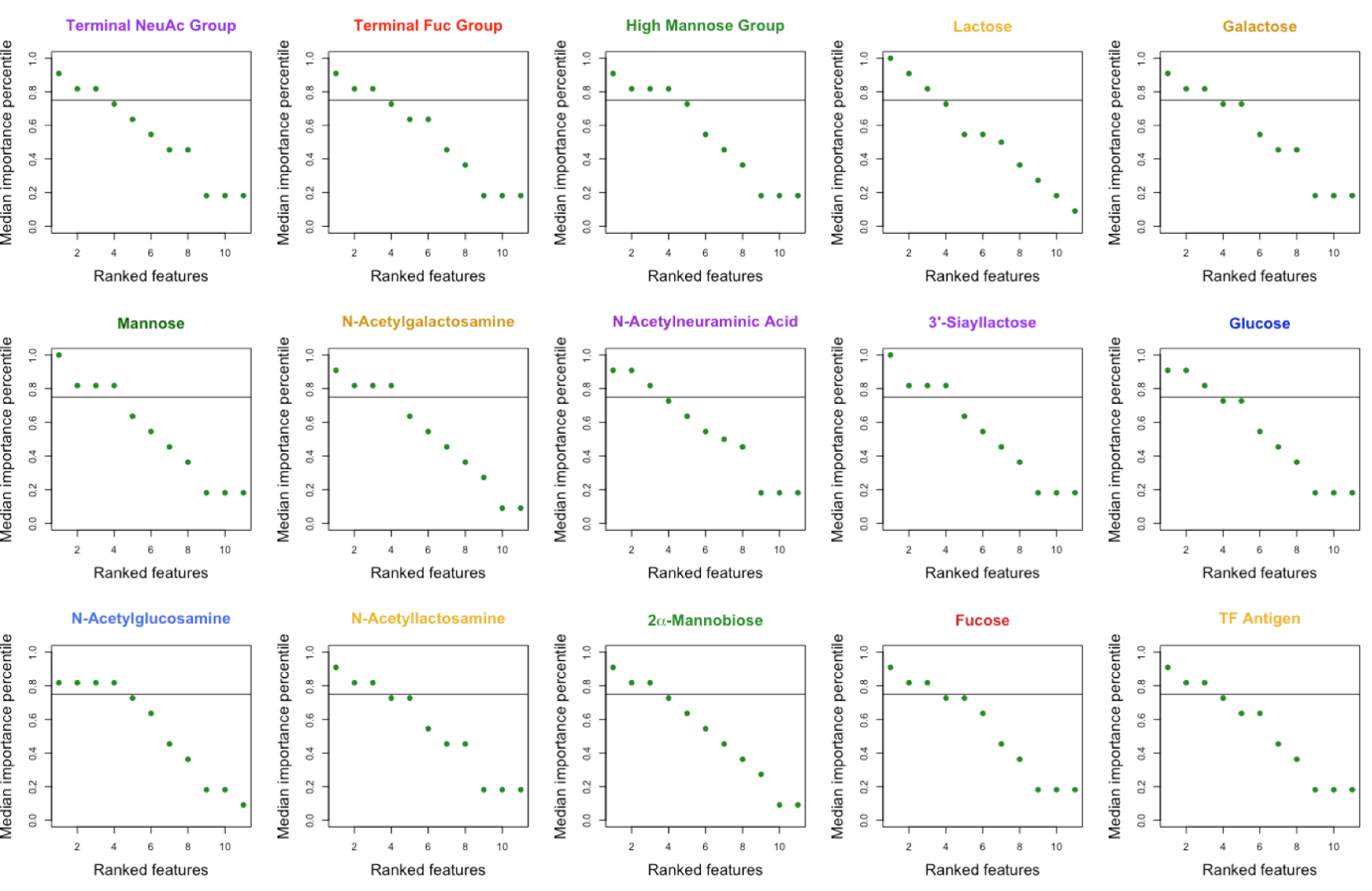

Supplement: S10 Fig — Median feature importance percentiles of PLIP features (within the PLIP features only) from each glycan-specific RF model determined via mean decrease in Gini impurity. Features with median importance in at least the 75th percentile (horizontal line) were considered highly predictive. Points were colored with the same color scheme detailed in Figs 1, 2, 4 and 6. (TIF) [file pcbi.1009470.s011.tif]

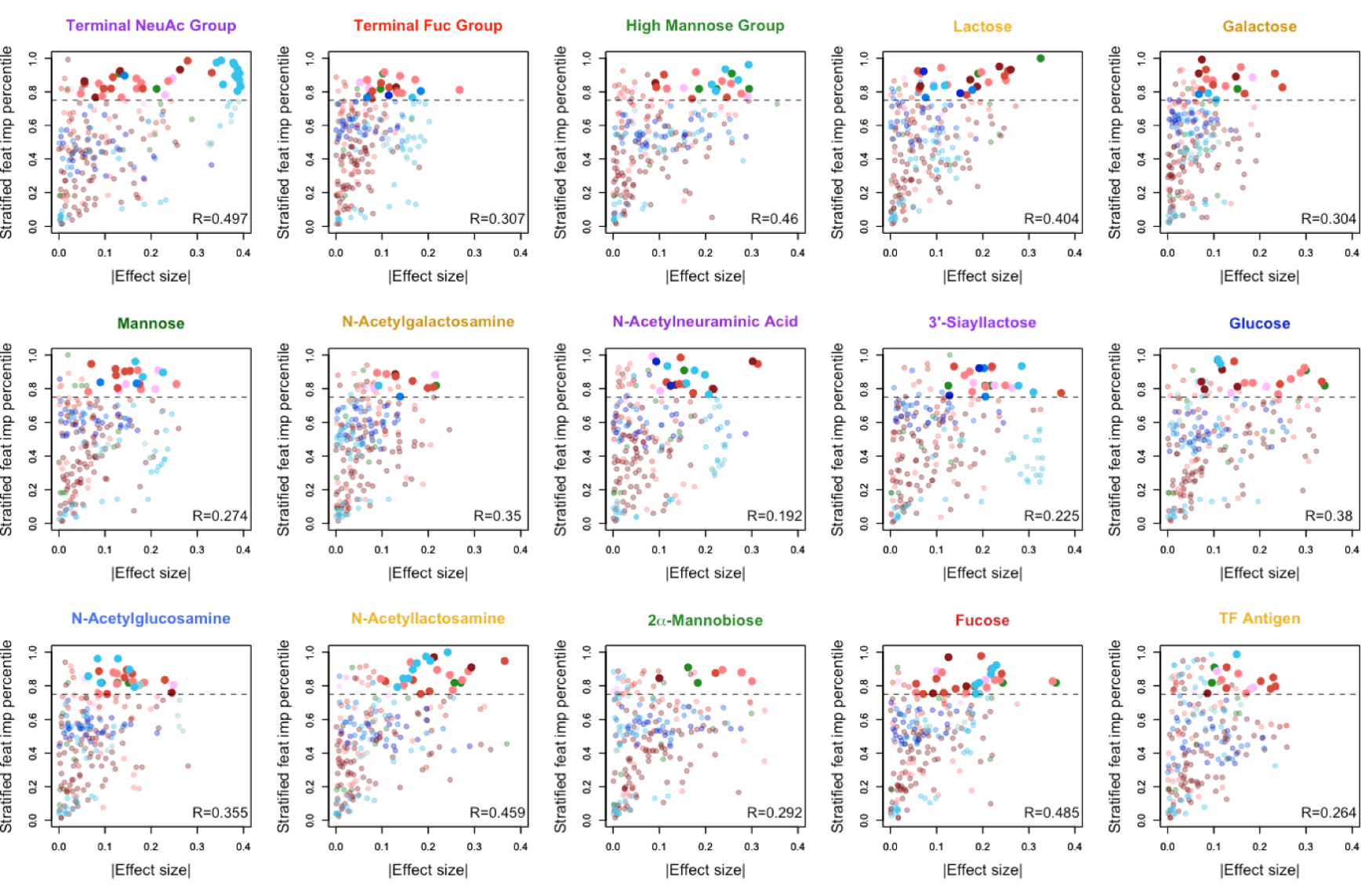

Supplement: S11 Fig — Median ranked feature importance percentiles (stratified by feature type) are plotted together against the absolute value of the weighted WMW effect size. In general, the stronger the observed association from the WMW test for a given feature, the more likely the feature was to be highly predictive. The dotted horizontal line indicates the 75th percentile threshold. Points that are bolded represent features that passed the 75th percentile for feature importance and were found to signficant from the weighted WMW test at q < 0.01 following the Benjamini-Hochberg procedure. Points were colored with the same color scheme detailed in Figs 1, 2, 4 and 6. (TIF) [file pcbi.1009470.s012.tif]

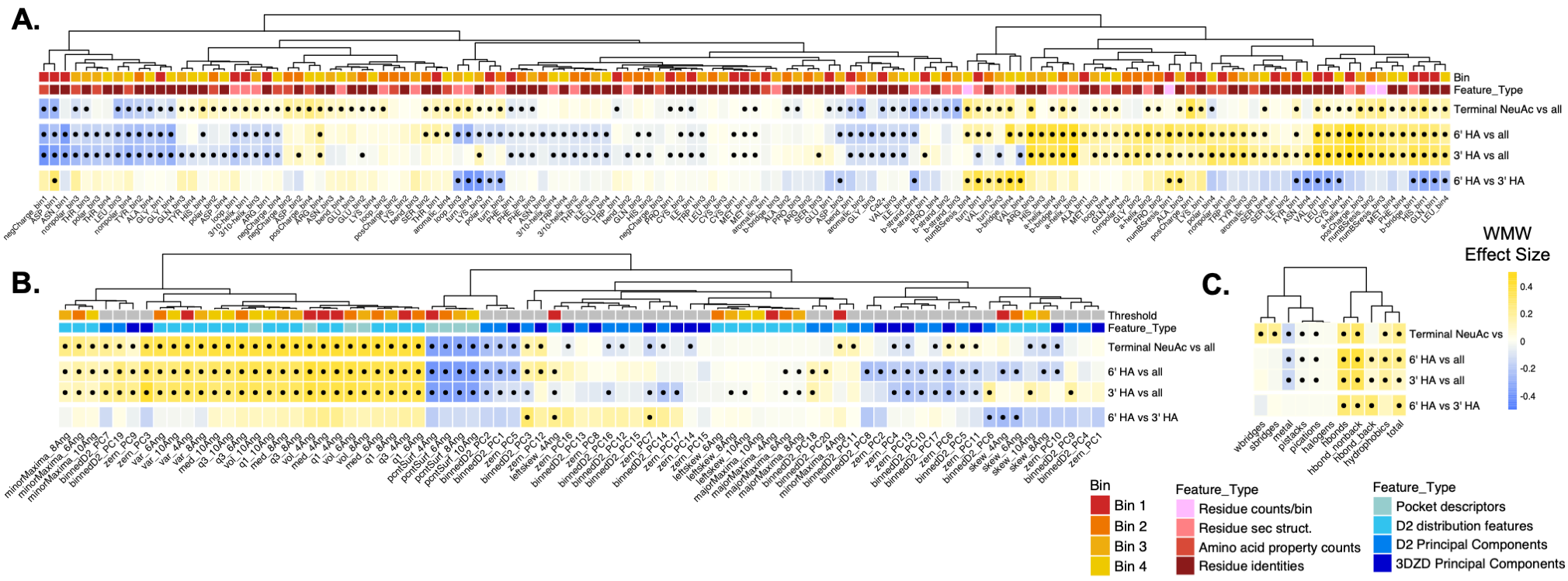

Supplement: S12 Fig — Enrichment and depletion patterns in the 221 features for 6’ NeuAc glycans compared to 3’ NeuAc glycans (bottom row) determined by a weighted WMW test. Bullet points indicate q < 0.01 by Benjamini-Hochberg correction. The first row shows the associations for terminal NeuAc glycans compared to background from Fig 4 for ease of comparison, and the second and third row show comparisons of 6’ NeuAc glycans and 3’ NeuAc glycans in HA binding sites compared to background interactions. (TIF) [file pcbi.1009470.s013.tif]

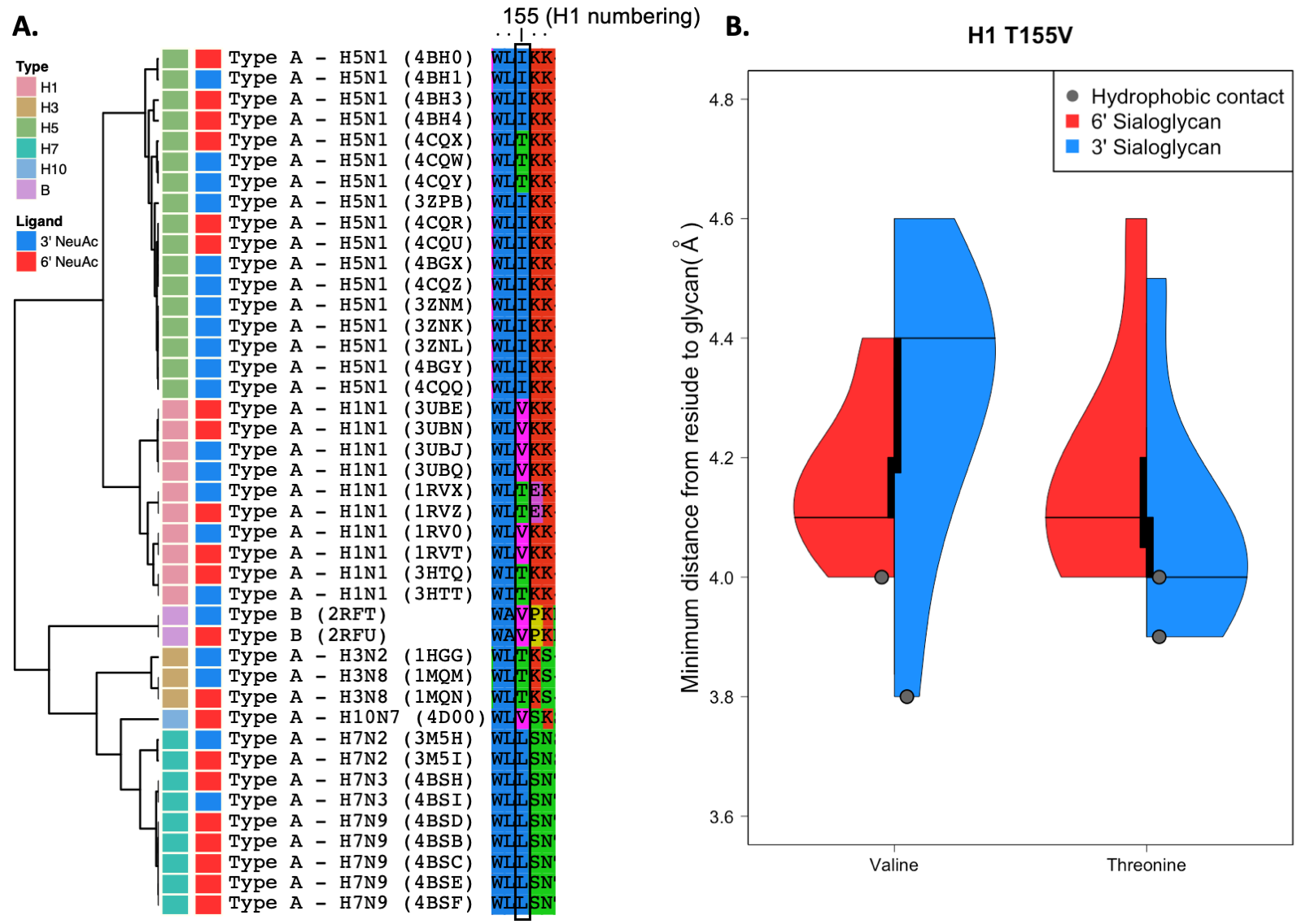

Supplement: S13 Fig — Valine appears at position 155 (H1 numbering) in H1, H10, and type B hemagglutinin structures, as shown by the multiple sequence alignment (Clustal Omega), visualized with Seaview, and clustered by global sequence identity (BLOSUM 62), as show in panel A. Panel B shows the distributions of the measured minimum distance from any atom in the residue at position 155 to the closest heavy glycan atom (usually the terminal carbon of the N-acetyl group) within all HA structures from H1N1. When complexed with 3’ sialoglycans, threonine is usually oriented closer to the glycan compared to valine, and has a hydrophobic contact with the sugar in two of the structures (compared to one structure when valine is present). When complexed with 6’ sialyoglycans, valine is more tightly grouped closer to the glycan and has one observed hydrophobic interaction with the glycan while threonine has no contacts. (TIF) [file pcbi.1009470.s014.tif]
