# Supplementary material for: Comprehensive analysis of lectin-glycan interactions reveals determinants of lectin specificity
Source: PLoS Comput Biol. 2021 Oct 6;17(10):e1009470. doi: 10.1371/journal.pcbi.1009470 (PMC8523061; doi:10.1371/journal.pcbi.1009470)
Supplement: S1 Table — (PDF) [file pcbi.1009470.s015.pdf]

1	NeuAc(a2-3)Gal(b1-4)Glc
2	NeuAc(a2-6)Gal(b1-4)GlcNAc
3	NeuAc(a2-3)Gal(b1-4)GlcNAc
4	NeuAc(a2-3)Gal
5	NeuAc(a2-6)Gal
6	NeuAc(a2-6)Gal(b1-4)GlcNAc(b1-3)Gal(b1-4)Glc
7	NeuAc(a2-3)Gal(b1-3)GalNAc(b1-4)[NeuAc(a2-3)]Gal(b1-4)Glc
8	NeuAc(a2-6)Gal(b1-4)Glc
9	NeuAc(a2-3)Gal(b1-3)GlcNAc
10	NeuAc(a2-3)Gal(b1-3)GlcNAc(b1-3)Gal(b1-4)Glc
11	NeuAc(a2-3)Gal(b1-3)[NeuAc(a2-6)]GlcNAc
12	NeuAc(a2-8)NeuAc(a2-3)Gal(b1-4)Glc
13	NeuAc(a2-8)NeuAc(a2-3)Gal
14	NeuAc(a2-6)GalNAc
15	NeuAc(a2-6)GlcNAc
16	NeuAc(a2-3)Gal(b1-3)GalNAc
17	NeuAc(a2-3)Gal(b1-3)GalNAc(b1-3)Gal(a1-4)Gal(b1-4)Glc
18	NeuAc(a2-8)NeuAc
19	NeuAc(a2-8)NeuAc(a2-3)Gal(b1-3)GalNAc(b1-4)[NeuAc(a2-3)]Gal
20	NeuAc(a2-3)Gal(b1-3)GalNAc(b1-4)Gal
21	NeuAc(a2-8)NeuAc(a2-3)Gal(b1-3)GalNAc(b1-4)Gal
22	NeuAc(a2-6)Gal(b1-4)GlcNAc(b1-3)Gal
23	NeuAc(a2-3)Gal(a1-4)GlcNAc
24	NeuAc(a2-6)Gal;NeuAc(a2-6)Gal(b1-4)GlcNAc
25	NeuAc(a2-3)Gal(b1-3)GalNAc(b1-4)[NeuAc(a2-8)NeuAc(a2-3)]Gal(b1-4)Glc
26	NeuAc(a2-8)NeuAc(a2-8)NeuAc
27	NeuAc(a2-3)Gal(b1-3)[NeuAc(a2-6)]GalNAc

S1 Table. UniLectin3D-assigned IUPAC glycan names within the terminal NeuAc group of glycans.
