# Supplementary material for: Comprehensive analysis of lectin-glycan interactions reveals determinants of lectin specificity
Source: PLoS Comput Biol. 2021 Oct 6;17(10):e1009470. doi: 10.1371/journal.pcbi.1009470 (PMC8523061; doi:10.1371/journal.pcbi.1009470)
Supplement: S2 Table — (PDF) [file pcbi.1009470.s016.pdf]

1	Man(a1-2)Man(a1-2)Man
2	Man(a1-6)[Man(a1-3)]Man
3	Man(a1-3)[Man(a1-6)]Man
4	Man(a1-6)[Man(a1-3)]Man(a1-6)[Man(a1-3)]Man
5	Man(a1-6)[Man(a1-3)]Man(a1-6)Man
6	Man(a1-2)Man(a1-3)Man
7	Man(a1-6)[Man(a1-3)]Man(a1-6)[Man(a1-2)Man(a1-3)]Man
8	Man(a1-3)[Man(a1-6)]Man(b1-4)GlcNAc(b1-4)GlcNAc
9	Man(a1-2)Man(a1-3)[Man(a1-2)Man(a1-6)]Man
10	Man(a1-2)Man(a1-3)[Man(a1-3)Man(a1-6)]Man
11	Man(a1-3)Man(a1-3)Man
12	Man(a1-6)Man(a1-6)Man
13	Man(a1-2)Man(a1-3)[Man(a1-6)]Man(a1-6)[Man(a1-2)Man(a1-2)Man(a1-3)]Man
14	Man(a1-2)Man(a1-6)[Man(a1-3)]Man(a1-6)Man
15	Man(a1-2)Man(a1-6)[Man(a1-2)Man(a1-3)]Man(a1-6)[Man(a1-2)Man(a1-2) Man(a1-3)]Man(b1-4)GlcNAc(b1-4)GlcNAc
16	Man(a1-2)Man(a1-3)[Man(a1-6)]Man

S2 Table. UniLectin3D-assigned IUPAC glycan names within the high mannose group of glycans.
