# Supplementary material for: Comprehensive analysis of lectin-glycan interactions reveals determinants of lectin specificity
Source: PLoS Comput Biol. 2021 Oct 6;17(10):e1009470. doi: 10.1371/journal.pcbi.1009470 (PMC8523061; doi:10.1371/journal.pcbi.1009470)
Supplement: S3 Table — (PDF) [file pcbi.1009470.s017.pdf]

1	Fuc(a1-2)Gal(b1-4)Glc
2	Fuc(a1-2)Gal(b1-4)[Fuc(a1-3)]GlcNAc
3	Fuc(a1-2)Gal(b1-4)GlcNAc
4	Fuc(a1-2)Gal(b1-3)[Fuc(a1-4)]GlcNAc
5	Fuc(a1-2)Gal(b1-3)GlcNAc(b1-3)Gal(b1-4)Glc
6	Fuc(a1-2)Gal(b1-3)GlcNAc
7	Fuc(a1-2)Gal(b1-3)[Fuc(a1-4)]GlcNAc(b1-3)Gal(b1-4)Glc
8	Fuc(a1-6)GlcNAc
9	Fuc(a1-2)Gal(b1-3)[Fuc(a1-4)]GlcNAc(b1-3)Gal
10	Fuc(a1-2)Gal(b1-2)Xyl
11	Fuc(a1-2)Gal(b1-3)GlcNAc(b1-3)Gal
12	Fuc(a1-4)GlcNAc
13	Fuc(a1-2)Gal(b1-3)GalNAc(b1-3)Gal(a1-4)Gal
14	Fuc(a1-2)Gal
15	Gal(b1-4)[Fuc(a1-3)]GlcNAc(b1-3)Gal(b1-4)Glc
16	Gal(b1-4)[Fuc(a1-3)]GlcNAc
17	Gal(a1-3)[Fuc(a1-2)]Gal
18	GalNAc(a1-3)[Fuc(a1-2)]Gal(b1-3)[Fuc(a1-4)]GlcNAc
19	Gal(a1-3)[Fuc(a1-2)]Gal(b1-3)[Fuc(a1-4)]GlcNAc(b1-3)Gal(b1-4)Glc
20	GalNAc(a1-3)[Fuc(a1-2)]Gal(b1-3)GlcNAc(b1-3)Gal(b1-4)Glc
21	GalNAc(a1-3)[Fuc(a1-2)]Gal(b1-4)[Fuc(a1-3)]Glc
22	GalNAc(a1-3)[Fuc(a1-2)]Gal(b1-4)[Fuc(a1-3)]GlcNAc
23	GalNAc(a1-3)[Fuc(a1-2)]Gal
24	Gal(a1-3)[Fuc(a1-2)]Gal(b1-4)Glc
25	Gal(b1-4)[Fuc(a1-3)]GlcNAc(b1-4)Glc
26	Gal(b1-4)[Fuc(a1-3)]GlcNAc(b1-3)Gal
27	Gal(b1-3)[Fuc(a1-4)]GlcNAc
28	Gal(b1-3)[Fuc(a1-4)]GlcNAc(b1-3)Gal
29	GalNAc(a1-3)[Fuc(a1-2)]Gal(b1-4)GlcNAc
30	GalNAc(a1-3)[Fuc(a1-2)]Gal(b1-4)Glc
31	Gal(b1-4)[Fuc(a1-3)]GlcNAc(b1-2)Man(a1-3)[GlcNAc(b1-2)Man(a1-6)] Man(b1-4)GlcNAc(b1-4)[Fuc(a1-6)]GlcNAc
32	Gal(a1-3)[Fuc(a1-2)]Gal(b1-3)GlcNAc(b1-3)Gal(b1-4)Glc
33	GalNAc(a1-3)[Fuc(a1-2)]Gal(b1-3)GlcNAc
34	Gal(b1-4)[Fuc(a1-3)]Glc
35	GlcNAc(b1-4)[Fuc(a1-3)]GlcNAc

S3 Table. UniLectin3D-assigned IUPAC glycan names within the terminal fucose group of glycans.
