# Supplementary material for: Comprehensive analysis of lectin-glycan interactions reveals determinants of lectin specificity
Source: PLoS Comput Biol. 2021 Oct 6;17(10):e1009470. doi: 10.1371/journal.pcbi.1009470 (PMC8523061; doi:10.1371/journal.pcbi.1009470)
Supplement: S4 Table — (PDF) [file pcbi.1009470.s018.pdf]

1	NeuAc(a2-6)Gal(b1-4)GlcNAc
2	NeuAc(a2-6)Gal(b1-4)GlcNAc(b1-3)Gal(b1-4)Glc
3	NeuAc(a2-6)Gal
4	NeuAc(a2-6)Gal(b1-4)GlcNAc(b1-3)Gal
5	NeuAc(a2-6)Gal;NeuAc(a2-6)Gal(b1-4)GlcNAc

S4 Table. UniLectin3D-assigned IUPAC glycan names within the 6' αNeuAc-terminal glycans complexed with influenza hemagglutinin.
