# Supplementary material for: Comprehensive analysis of lectin-glycan interactions reveals determinants of lectin specificity
Source: PLoS Comput Biol. 2021 Oct 6;17(10):e1009470. doi: 10.1371/journal.pcbi.1009470 (PMC8523061; doi:10.1371/journal.pcbi.1009470)
Supplement: S5 Table — (PDF) [file pcbi.1009470.s019.pdf]

1	NeuAc(a2-3)Gal(b1-3)GlcNAc(b1-3)Gal(b1-4)Glc
2	NeuAc(a2-3)Gal(b1-4)GlcNAc
3	NeuAc(a2-3)Gal(b1-4)Glc
4	NeuAc(a2-3)Gal
5	NeuAc(a2-3)Gal(a1-4)GlcNAc
6	NeuAc(a2-3)Gal(a1-4)[Fuc(a1-3)]GlcNAc
7	NeuAc(a2-3)Gal(b1-3)GlcNAc

S5 Table. **UniLectin3D-assigned IUPAC glycan names within the 3' αNeuAc-terminal glycans complexed with influenza hemagglutinin.**
