# Supplementary material for: Comprehensive analysis of lectin-glycan interactions reveals determinants of lectin specificity
Source: PLoS Comput Biol. 2021 Oct 6;17(10):e1009470. doi: 10.1371/journal.pcbi.1009470 (PMC8523061; doi:10.1371/journal.pcbi.1009470)
Supplement: Attachment — Submitted filename: response.pdf [file pcbi.1009470.s020.pdf]

We wish to express our gratitude to the editors and reviewers for their consideration and insights, including their generally positive feedback on the value of our approach to characterizing lectin specificity. We have taken the reviewers' comments under very careful consideration and hope that the revised manuscript addresses all of their concerns. We respond to each comment individually below but want first to briefly respond to some of the common themes across the reviews, while summarizing some key points regarding our approach and analyses that, based on this exchange, we have sought to clarify in the revised manuscript.

The suggestions from multiple reviewers to re-evaluate the terminal NeuAc group and terminal fucose groups of glycans was well received, and the glycans comprising these groups have been re-evaluated such that the terminal NeuAc group only contains realistic, existing glycans without fucosylation and the terminal fucose group contains non-sialylated glycans with a fucose terminal position either as a alpha1,2 linked fucose or as a branched "arm" stemming from a residue towards the non-reducing terminal end of the glycan structure. The shifts in these glycan classes did not substantially impact the results, but it is reassuring to know these groups are now more appropriately defined.

There were common questions or misconceptions about our goals and approach, and in the revision we have worked to ensure that these are clearer. In summary, we present a novel approach to investigating lectin specificity, via comprehensive analysis of features in crystal structures of glycan-bound lectins. We demonstrate the utility of this type of analysis by characterizing both how the features of lectins binding one glycan (or group) differ from those binding others (one-vs-others, or *global* specificity; sections 2.2-2.4 / figures 2-5) and how the features of lectins binding very similar glycans differ from each other (some-vs-some, or *fine* specificity; section 2.5 / figure 6). In global specificity, we employ both comparative and predictive analyses. Importantly, each glycan is studied separately as the "one" in one-vs-others, as we seek to investigate what distinguishes it from all the others; we do not attempt to build a single unified predictive model that simultaneously distinguishes each from each other. For fine specificity, the data was only sufficient to support comparative analyses (i.e., there were too few examples to establish rigorous training and validation sets while controlling for homology), and here we characterize differences in alpha2,6-linked NeuAc versus alpha2,3-linked NeuAc glycans. We elaborated both the introduction and results text, along with Figure 1E, to try to ensure clear distinctions between goals and methods for global and fine specificity.

Overall, we seek to demonstrate a novel, general "pattern" of approach for analyzing lectin-glycan specificity based on structural features. On the one hand, lectin specificity is definitely more nuanced than we could hope to detail here for any individual lectin, and on the other hand, it would be very powerful to have an overarching model predicting binding preferences of all lectins and glycans. Given the data at hand, we arrived somewhere between these aspirations, but feel that our approach is still able to serve as a proof of principle and potential roadmap for further structural studies of lectin specificity.

We now turn to the individual comments.

Reviewer #1

In this manuscript the authors present an extensive statistical classification of lectins from the UniLectin database with the aim of determining the fundamental structural and electrostatic/hydrophobic characteristics driving molecular recognition of specific glycan epitopes and monosaccharides. This classification is based on 221 features, the authors carefully selected as essential determinants driving binding. In my opinion this work is timely, and the aims address a very important matter, which solution would indeed open the field for the use of lectins in carbohydrate sequence, structure, and binding characterization. For this reason, I'd like to congratulate the authors for embarking in such interesting and potentially critical study for the advancement of glycoscience, and I hope the suggestion below will help improve not only the readability of the manuscript itself, but also (and more importantly) the predictive potentials of the method.

We thank the reviewer for this summary and positive feedback on this line of research.

Indeed, after carefully reading the manuscript, I unfortunately did not find the results convincing, especially in terms of the ability of the method to predict the binding specificity of lectins. More specifically, the attribution of enriched and depleted features, which should be a discriminant for glycan specificity is unclear and in my opinion, heavily biased throughout by the prior knowledge of the preferential lectins' binding epitope, so extremely hard to generalize for a blind prediction.

We are unfortunately not entirely clear on this critique, though we think that perhaps it stems from the reviewer's desire to see a "grand unified" model of lectin specificity -- a desire that we share, but, as discussed above in the overarching comments, is not the goal of the present study. By pursuing a structure-based analysis of glycan-lectin binding, we are naturally dependent on the available crystal structures. But our aim here is to illustrate that there are significant differences between the structural features of pockets accommodating each given glycan individually vs. those accommodating others. We take substantial care to control for the most significant biases that might affect the interpretation -- redundancy (or even substantial similarity) among sequences and structures. For characterizing determinants of global specificity, we use both standard comparative analyses (enriched and depleted features), as well as predictive modeling (one-vs-other), in each case carefully demonstrating the significance (statistical tests) or robustness (comparison to background models) of the approach. Thus we believe that the results indeed provide a strong, quantitative basis for confidence in their generalization ability, within the context established (i.e., not blind prediction in a unified model).

In the revised manuscript, we have sought to further clarify the scope, goals, and limitations of the approach and conclusions, along these lines.

As a demonstration of that, in my view the test case on HA illustrates a problem, as the HA binding site does not satisfy the characteristics the authors have highlighted as distinctive for a terminal sialic acid specific site, namely there is no high density of positively charged (at pH 7) residues, but actually an increase in negatively charged residues associated to the change in specificity from alpha(2-6/3), and the binding site is structurally quite shallow (no pockets). Therefore, the method may not identify the latter as a sialic acid binding site.

We appreciate this interpretation and hope clarifications in the scope and execution of the analysis might assuage these concerns. In particular, the highlighted characteristics of lectin interactions with terminal sialic acid were from the one-vs-others global specificity analysis, distinguishing these interactions from all other lectins with all other glycans. In contrast, the characteristics shown by the enriched and depleted features in Fig 6B are from some-vs-some fine specificity analysis, characterizing alpha2-6 binding sites compared to alpha2-3 binding sites. The increase in negatively charged residues is known to be associated with alpha2-6 specificity from literature as referenced in manuscript (E190D & G220D in H1 binding sites).

Relative to background lectin binding sites, HA binding sites are quite similar regardless of alpha2-3 vs alpha2-6 fine specificity. Both the representative interactions in Fig 6 panels C & D have pockets with fairly substantial volume; the voxelized pocket representations were not shown for the sake of simplicity but for added clarity, they have been added in as subset panels as done in Fig 5. We have updated supplemental figure 12 to show features enriched/depleted in HA-a26 interactions compared to background, as well as for HA-a23 interactions compared to background, demonstrating these sites are quite similar to characterization of terminal NeuAc-recognizing binding sites.

The goal of the fine specificity analysis is primarily to demonstrate that even though lectin interactions with very similar glycans might appear almost identical in a global specificity analysis comparing them to background interactions, comparing them to each other allows for focused investigation into the differences that do exist. We have elaborated the introduction and results around this fine specificity analysis to further discuss the complementary goals of global and fine specificity.

I believe that the problem may rest with the choice of glycans ‘categories’ used to train the method.

We want to reiterate that in our analyses, there is not a singular model trained across all glycans (or glycan categories), but rather a separate model for each glycan (or glycan category) derived by following the same approach, with the goal of uncovering insights into each specificity individually.

For example, terminal sialic acids in the same group are either 2-3/6, there are also polysialic motifs with a terminal 2-8, also in the same group. Some of those sialylated glycans also contain fucose in the arms (LeX) and core fucose. Within this context it’s important to underline that terminal Sia in complex N-glycans is always linked to a LaNAc or to other structures in other glycans, so that it never comes alone and the binding site has evolved to form specific interactions along the disaccharide/trisaccharide 3D motif. Therefore, it is not reasonable to expect different features to play a role in selecting either terminal sialic acid or LacNAc, as both may be bound in the same site.

We agree that it is unreasonable to expect completely independent and separate associations for these features, especially since a non-trivial number of the examples come from the same lectins with different ligands. This point is referenced in the first paragraph of section 2.3.

Indeed, this gets back to the global vs. fine specificity distinction, and motivates the work shown in section 2.4/figure 4 & 5 to examine shared similarities and differences compared to background lectin binding sites for each glycan/group of glycans. There are indeed some striking similarities between interactions with Lac/LacNAc and with the terminal NeuAc glycans. This has been noted in the revised manuscript and we thank the reviewer for suggesting this confirmatory result.

Also, the generalization to terminal fucose is a bit ambiguous, as most of the chosen epitopes all contain 1-2 fucose, not terminal fucose in the arms (LeX) nor core fucose, which are (strictly speaking) both ‘terminal’ as nothing is linked to them.

We thank the reviewer for pointing out the ambiguity in this group of glycans and have expanded the Terminal Fucose group in Table S3 to include 21 additional non-sialylated glycans with terminal fucose in the arms, and have updated the figures and text to reflect the new results for the terminal fucose category.

As a suggestion that the authors may find useful, in my opionion the algorithm could benefit from a better-refined choice of the glycans substrates, so that they constitute uniform groups, consistent in terms of sequence and structure. Both these characteristics highly affect the 3D structure of the epitope, making it unique, which is fundamental as the lectin binding site specificity hinges on a structural and electrostatic complementarity to the epitope, that ultimately allows for recognition and binding.

We wish to again express that our work does not represent a singular algorithm but rather a thorough analytical approach applied in parallel for different glycans. The choice of glycan ligands for one particular application of the analysis approach is certainly important, but it primarily informs the interpretation of the results for that individual set of glycan ligands but not the general utility of the analysis approach or the conclusions regarding other glycans.

We absolutely agree that more uniformity in the glycan ligands would be very beneficial, but we are limited by the quality of the glycan structures in the PDB (discussed on lines 540-545), and as a result even within single groups of glycans/monosaccharides there is still structural variation in terms of modifications of residues and what portion of a glycan is actually resolved in a crystal structure. Significant effort has gone into tools to verify structures and glycan identities in the PDB but we could not find any suitable for our needs (mass characterization of 4000+ structures) and this is beyond the scope of what we could accomplish. As such, we relied on manually annotated glycan identities from UniLectin which are great but still imperfect.

The use of the 13 different glycans that are sufficiently well-represented across diverse lectin structures was our best attempt to focus our results on uniform, consistent groups. The additional investigation into the three groups of more diverse glycan structures was included to demonstrate broader applications of this approach and the ability to recover associations despite increased glycan diversity.

For example, Sia(2-3)LacNAc has a different structure relative to Sia(2-6)LacNAc, as it can be seen in the figures illustrating the HA test case; some lectins do bind either, but a general

preference or promiscuity can indeed be revealed by a method trained to select for each of those separately, which would be incredibly powerful.

The differences in glycan structures/orientations is well-noted, and is discussed in lines 479-481, 533-535 & 545-552 in the revised manuscript. We have elaborated on this point in the revised manuscript for added clarity.

More broadly, we agree entirely that more advanced models and studies of fine lectin specificity would be very powerful and hope this study demonstrates the existence of features that could be used to this end, especially using more appropriate and detailed assessments of specificity such as the modified sialic acid microarray from the NCFG. However, a significant amount of work would be required to build more holistic lectin binding site representations that are robust to differences in the size and orientation of crystallized glycans (as mentioned above) and to obtain the microarray data for sufficient lectins to have statistical power to build such a method. We raise this point in the discussion section as a valuable direction for future research.

Moreover, in this matter, I found unclear how a method not trained to distinguish 2-3 from 2-6 sialic acid can indeed predict changes in specificity in HA, and especially that an increase in negatively charged residues (which as mentioned earlier, goes against the described/reported characteristics of a terminal sialic acid-specific binding site) is associated to 2-3 preference.

As discussed in the overarching comments (and reemphasized in the results in the revision), due to data limitations, predictive classification was only performed for the global specificity analysis and not for the fine specificity analysis brought up by the reviewer. For fine specificity, the featurized representations of the binding sites containing each glycan were objectively compared to each other in a comparative approach. While an increase in negatively charged residues compared to 2-3 NeuAc binding sites was seen in 2-6 NeuAc binding sites, this change corresponds to known mutations in literature that play critical roles in shifting HA specificity (E190D & G220D) as discussed in a previous point. The observed increase in negatively charged residues is compared to 2-3 residues, while the general depletion of negatively charged residues seen in lectin interactions with sialic acid containing glycans was seen compared to all other lectin binding sites. The revised supplementary figure 12 further demonstrates this point, showing negatively charged residues are significantly depleted compared to background interactions for both 2-3 and 2-6 bound hemagglutinin. We hope the revised manuscript makes this more intuitive.

Another question I could not find the answer to and that the authors may consider addressing/clarifying is, how was the specificity to isolated monosaccharides assessed? I am not aware of lectins binding isolated monosaccharides, such as fucose, sialic acid, i.e. not in a context of a larger glycan.

Specificity of lectins to singular monosaccharides was assessed in the same manner as specificity to larger glycan structures: co-occurence in crystal structures in the PDB. While it is also our understanding that lectins do not typically bind individual monosaccharides, that is not always the case (seen in the recognition of O-GlcNAcylation by AAL2 and discussed in lines 412-415. Additionally, monosaccharide-level specificity was the primary way in which lectin

(agglutinin) specificities were defined historically through competitive inhibition of agglutination experiments with different monosaccharides (lines 58-60 & ref. 27)

We acknowledge in the discussion that this is a primary limitation of our study and hope this proof-of-concept will inspire further study into this question with more detailed characterizations of lectin specificity. At present, the primary limitation is succinctly defining lectin specificities in a manner that allows for straightforward comparisons and potentially predictive classification, as well as relating studies of specificity back to individual lectins/crystal structures while accounting for subtle differences in the binding patterns of the “same” lectins from different sources (native vs recombinant or different vendors).

As a few minor points, I found the manuscript a bit too long (50 odd pages) and discursive. In my opinion, most of the method details could easily go into supplementary material, where the interested reader can find them, without disrupting the flow.

As this approach is relatively novel, especially in the lectin/glycan space, we wanted to be thorough and clear in the methods we applied and considerations we made. We attempted to limit methodological discussion outside of the Methods section to what was necessary for contextual understanding of the results. However, if the editor so guides, we would be happy to move some of the methods section to a supplementary file as appropriate.

Some of the graphs and figures are too difficult to read as the text is very small, e.g. Fig 4 and 6A.

We thank the reviewer for this feedback and acknowledge this as an issue, as the figures do contain substantial content. In figure 4, we have increased the font size of the row labels and column color bars in all panels. In figure 6, we have increased the text size of all legends, the color scale label, and the color bar labels. In both figures 4 & 6, the individual row and column labels are as large as possible without overlapping and becoming completely illegible. We recognize this text is likely too small to be easily read and as such, the column and row color bars contain the same basic information to facilitate understanding of the figure and to allow interested readers to zoom in on specific regions for a closer look. Unfortunately, this text can’t feasibly be made larger in the current layouts. Alternatively, this text could be removed in the main manuscript and the figures might be recreated in the supplemental material with larger row and column labels if this font size is totally unacceptable.

An additional feature not considered and that I believe may be useful is the lectin’s fold.

At one point, we did consider incorporating fold, but ultimately decided it would be more interesting to exclude it, and focus only on the features of the binding pocket and not the “scaffold” in which the pocket appears. This can also allow for differences in pockets within the same lectin. Our primary goal was to identify physicochemical and geometric associations with lectin specificity, ideally independent of the fold/scaffold carrying the binding site even though some information related to the nature of the entire lectin fold would naturally still be captured in the features with larger thresholds/binned distances. In subsequent work where predicting lectin

specificity with the highest possible accuracy is the goal, lectin fold is likely useful information to include and we include in the discussion this suggestion for future efforts in this area.

Line 398, consider replacing with N-Acetyl Glucosamine and Galactosamine or GlcNAc and GalNAc.

We thank the reviewer for this suggestion and have reworded this heading. We had previously gone back and forth on the best way to phrase this; hopefully this revision is clearer.

Reviewer #2

The manuscript describes a novel bioinformatics analysis of 3D structures of lectin binding sites, with the aim of deciphering the structural basis of specificity and to obtain a predictive tool. The work is clearly described and the results are of high interest since clear separation could be obtained between different classes of specificity. Application to the fine specificity of hemagglutinins towards different sialylated oligosaccharides reached the prediction level. Altogether, it is a very original approach that will benefit to the glycobiology community.

We thank the reviewer for this nice summary and positive feedback. As mentioned in the overarching comments, we do wish to clarify that predictive analysis was performed for global specificity but not for fine specificity of influenza hemagglutinin. The revised manuscript hopefully more clearly outlines the analysis performed.

Some points need to be clarified, or revised, in relation with the complex behaviour of lectins that may complicate some of the interpretation.

Main points

- Lectins recognize spatial arrangement of hydroxyl groups (and N-acetyl or methyl) that can be generated by different scaffold in a rather similar way. NeuAc and GlcNAc can be recognized by same lectin (WGA, PVL …) with different orientation of the ring. Same with Fuc and Man (LecB)… Did the authors considered crystal structures of one lectins with different ligand when appropriate ?

This is an interesting question. We did look for overlap between feature enrichment for mannose and fucose monosaccharides but did not see any clear patterns beyond some similar geometric feature associations. And unfortunately, with binding site representations informed by the specific glycans being bound, we did not have a straightforward way to compare different examples of the same lectin with different ligands. We raise this important question in the discussion in the hope of pointing future studies toward addressing it.

- The discussion about differences between mono/disaccharides and larger glycans bottom of page 22 is problematic. First, the term “conformational freedom” of monosaccharides should be replaced by “orientational freedom” . Second, the function of lectins is not to bind monosaccharides (opposite to transport protein) but to attach to complex glycan on surfaces.

The fact that many are co-crystallized with monosaccharide is a bias from experiments (easier to obtain crystals). So the differences should be rather analysed in term of binding to branch structures (lewis, oligomann) versus linear ones (sialylated)

We appreciate this suggestion and have changed the text to read “orientational freedom.”

We are in agreement on the second point, that the function is to recognize complex glycans. However, crystal structures are largely limited to monosaccharides. The purpose of our discussion was to highlight a potential limitation of the data consisting primarily of smaller glycans that are less functionally relevant to lectins, with a note as to how the differences in the sizes of crystallized ligand influenced the characterization of the binding sites. We have moved this sentence to a more relevant place in the paragraph to apply to discussions of the glycan orientations on interaction characterization and hope this is more clear**.**

It would be interesting to consider branched vs linear glycans with more robust definitions of the extent of the binding site, but in this implementation it is likely that observed associations would be dominated by shifts in the regions of the lectin considered as a binding site, the point originally intended to be addressed here.

- The separation between terminal sialylated and terminal fucose (Table S1 and S3) poses the problem of Sialyl Lexis x (NeuAc(a2-3)Gal(b1-4)[Fuc(a1-3)]GlcNAc that is listed as “terminal NeuAc” and not as “terminal Fuc” , while it can be bound by the Fuc moiety in some lectins

We thank the reviewer for bringing this point to our attention and we have omitted ligands 2, 15, 18, 30, and 37 from the Terminal NeuAc group as previously listed in Table S1 as they contain fucose residues which might obscure the analysis.

- The terminal Fuc compounds in Table 3 does not include Lewis x Gal(b1-4)[Fuc(a1-3)]GlcNAc that is a very common ligand in lectins / this should be checked

We thank the reviewer for pointing this out, and have expanded the Terminal Fucose group listed in Table S3 to include 21 additional non-sialylated glycans with fucose branches as they are also “terminal” but do not overlap with the Terminal NeuAc group.

- As pointed by the authors, there are some errors in Unilectin3D, and it would be better not to reproduce there in the present article. Ligands 12, 16 and 37 of Table 1 do not exist (likely typing error) and should not be included (corrections has been requested in unilectin3D, so likely to be corrected now). Also not sure about meaning of ligand 33

We thank the reviewer for noting this, and have omitted ligands 12, 16, and 33 as suggested, along with ligands 27, 32, and 36, from the Terminal NeuAc group as previously listed in Table S1 as their meaning is ambiguous and might not align with the intended definition of this group of glycans. Ligand 37 of Table S1 appeared realistic but was omitted (as mentioned above) to avoid fucose-containing glycans.

Details to be corrected

Authors summary

Line 10 : glycans are attached to proteins and lipids

Line 12 : should be “influenza virus”

Line 14 : “sweeter” is catchy but not appropriate : higher affinity ?

We thank the reviewer for listing these details to be corrected and have changed them in the author summary of the manuscript.

Introduction :

Line 24 ; :lectins are proteins, not protein domains (they have carbohydrate binding domain and other ones)

Line 29 : hemagglutinin lectin : “lectin” should be removed

We thank the reviewer for identifying these details to be corrected and have changed them in introduction of the manuscript.

Legend of Figure 1 : The complex selected for illustration does not include “bacterial lipopolysaccharide” but only a disaccharide fragment. The E plot needs some more description . What is the y-axis (definition, values), what is the color coding of the x-axis ?

The caption of the figure 1 has been updated to clarify the ligand shown in these structures.

We apologize for the ambiguity in the schematic representation of results in panel E of figure 1. We have revised panel E into two separate panels to distinguish independent parts of the analysis and attempted to add appropriate levels of description and detail.

Reviewer #3

The manuscript “Comprehensive analysis of lectin-glycan interactions reveals determinants of lectin specificity” aims to develop a systematic study to highlight complementary physiochemical and geometric features which allow to define the specificity of the lectin-glycans binding. After a general overview about the state of the art and considering the advantages and limitations of the experimental and computational techniques used in the field, the authors introduce the open points that they aim to address in this paper.

In this study, the authors screen and choose over 1300 structures, representing more than 400 lectins in complex with 226 glycan ligands, clustered at 50% of identity. The resulting 225 clusters were in subjected to interaction weighting and sampling based at each step of the analysis to prevent disproportionated impact from better-represented lectins clusters. To define the specific features defining the lectin-glycans binding, the authors adopt the univariable comparative analysis with weighted Wilcoxon-Mann-Whitney test which reveals specific lectin binding pocket features, and the multivariable modeling with random forests in combination with the previous analysis which demonstrate that in specific cases (i.e. NeuAc terminal glycans,

mannose monosaccharides and fucose monosaccharide) particular features combinations suffice to predict specific recognition. By integrating the 221 features as well as different type of features (i.e. pocket features, physicochemical environments and recognition motifs, 3D geometry based relationship, similarities in PLIP-characterizes atomic interaction) identified from the comparative and predictive approaches, the authors extracted global determinants of specificity that can be significant and predictive to identify the binding of similar glycans. Finally, the systematic analysis developed is adopted to successfully characterize the specific features which define the binding specificity between 3' and 6' α-NeuAc terminal glycans. Interestingly, the analysis not only recovers known mutations which drive the specificity of the above-mentioned binding but also uncovers a new potential physicochemical determinant for the 6' α-NeuAc terminal glycan specificity.

Overall, the work is carefully carried out and clearly described. It represents a useful systematic study that increases knowledge of lectin-glycan binding features laying the basis to obtain further insight into the field.

We thank the reviewer for this nice summary and positive feedback.

I however suggest the following minor edits about the study:

- Figure 1. In panel B, the code-colour should be the same as for the other panels. In panel C, clarify the role of the additional components. Overall, I suggest to make the workflow of the analysis easier to handle by clarifying that the data in Fig.1 E contains the features from Fig.1 B-C-D. In addition, it is not clear to me why the legend in Fig.1E (squares under the histograms) does not follow the same order of the Figures (B-C-D in figures VS B-D-C in legend).

We appreciate these suggestions and have updated the color of the structure in panel B to match the other structures. Additionally, we have added clarifying text in the figure to describe the additional components in the panel describing the 3D features. The order of the legend in panel E was set to match following figures but the subpanels were inconsistently ordered and have been re-ordered to be consistent throughout (along with descriptions of the features on page 6).

Based on feedback from all reviewers, panel E appears to have been unclear. We have revised it, splitting it into two separate panels to distinguish between the two separate components of the analysis (global and fine specificity, as discussed in the overarching comments) and provide better intuition for the following results in the manuscript.

- Line 200. Is it glucose or galactose? It is not clear to me why the authors choose the glucose because, in my opinion, the trend of the galactose seems more similar with the other mentioned. Can you explain it?

In this group, we attempted to highlight the glycan ligands with the strongest statistical associations (both positive and negative). This text has been slightly revised to state this more explicitly.

This approach is (obviously) somewhat subjective. The listed grouping is based on both the number of significantly associated features as well as the effect size. We have added thin vertical grid lines at the +/- 0.2 & 0.4 tick marks in Fig 2 to make it easier to compare effect sizes. It is hopefully clearer that glucose has many significant features with effect sizes more extreme than +/- 0.2, while galactose has only 2-3 significant features with effect sizes more extreme than +/- 0.2.

- Line 204. As previous, in my opinion LacNAc seems to have a similar trend as NAcetylneuraminic acid and 3' sialyllactose. Can you clarify it?

As discussed above, these observations are subjective. It was originally intended to indicate that LacNAc appears to have reduced associations relative to Lactose, with the difference further exaggerated in the case of TF antigen. A similar trend can be seen between the Terminal NeuAc group having far more features with more extreme effect sizes than NeuAc monosaccharide or 3'-SLN, likely attributable to similarities of the interactions and the number of highly similar interactions appearing in the background.

We have edited this section to hopefully clarify this storyline while keeping discussion streamlined as these relationships are explored more thoroughly in section 2.4.

- Figure 6B. I would suggest to help the reader following the discussion in association with the figure by highlighting the point discussed with arrows or something like that.

We thank the reviewer for this suggestion and have highlighted points in the volcano plot (6B) directly referenced in the text. To this end, we have also more clearly labeled the residues at position 155 in panels C and D of this figure.
