# Supplementary material for: Comprehensive analysis of lectin-glycan interactions reveals determinants of lectin specificity
Source: PLoS Comput Biol. 2021 Oct 6;17(10):e1009470. doi: 10.1371/journal.pcbi.1009470 (PMC8523061; doi:10.1371/journal.pcbi.1009470)
Supplement: Attachment — Submitted filename: response2.pdf [file pcbi.1009470.s021.pdf]

We would like to thank the reviewers and editors for their time and consideration in the careful review of this manuscript and are very appreciative of the constructive feedback we have received throughout the process. We are glad that the reviewers were generally satisfied with the revisions we made previously, and likewise we were happy in this round to make the further minor revisions suggested by the first reviewer. In summary, we have added a sentence to the abstract to acknowledge broad limitations covered in more detail in the discussion, clarified the statistical threshold used to determine statistical significance of feature associations (Fig 2 caption), and revised Fig 6 (and its caption) to remove the illegible labels that were not necessary to support interpretations in the results.

Reviewer #1: I'd like to thank the authors for their answer to my questions and for the modifications they made to this version of the manuscript, which in my opinion is a strong improvement over the original submission. I particularly appreciated the fact that the authors explained much better to the reader the limitations of their method (see more below), which stems from the (relatively low) number, diversity and degree of completeness of the structural data available to date in UniLectin. Also, the HA case is not framed anymore as a proof of concept, at least in my opinion it isn't, yet as a useful test of how looking at the selected features within the framework of a specific comparison between highly similar lectins, can indicate epitope specificity. I find this information extremely valuable to the whole community.

We thank the reviewer for their initial suggestions and are glad that they find our previous revisions to have substantially improved the manuscript. We also appreciate the statement of value to the glycobiology community and are hopeful it will be shared by readers.

I have only minor suggestions to this version that I'm hoping the authors will consider taking on board, as those will take very little time and effort, hopefully.

The comments and points about the limitation of the method are now made clear in the Discussion section, so reaching the "really" interested readers, i.e. the one who go through all the results. Tp the broader readership's benefit, I would suggest to add a sentence to that effect in the abstract.

We thank the reviewer for this suggestion and have added to the abstract an acknowledgement of limitations due to our reliance on lectin-glycan co-crystal structures.

Also, I find it would be important to indicate quantitatively what is the threshold for "statistical significance" used in the data presented in Figure 2, see caption. The authors could also refer

the reader to the method section, where those thresholds could be discussed in detail, if needed.

We used a significance threshold of $q<0.01$ to control the FDR at approximately 1%. We have revised the caption for figure 2 to indicate this.

I again recommend for the method to appear in the SI, or to shorten the manuscript some other way, yet the latter may be more time consuming.

While we are open to relegating the methods to the SI following the guidance of the editors, we advocate keeping the methods in the main text for easier reference while reading the manuscript and to ensure that the information important for thoroughly digesting the results is self-contained. Especially since this is an electronic publication without physical space constraints, it seems worthwhile to preserve the cohesiveness of the core text as a single file.

Figure 6 panel A is unreadable, I understand perfectly well that those details/labels cannot be made larger, but if they can't be read, they are hardly useful, in my opinion. That panel can maybe be transferred to SI and blown up?

We appreciate this feedback and agree that labels are not useful if they are illegible. In fact, we realized that the labels are not important to understanding the results; the information is fully contained in the color bars. We have thus removed the labels from figure 6 panel A, further allowing for more room to display these color bars.

Reviewer #2: The authors clarified all the requested points.

We thank the reviewer for their expertise and previous input on improvements to this manuscript.

Reviewer #3: Overall, the work is carefully carried out and clearly described. It represents a useful systematic study that increases knowledge of lectin-glycan binding features laying the basis to obtain further insight into the field. The minor edits requested have been satisfied. Based on this, I suggest the publication of the paper.

We thank the reviewer for their expertise and previous input on improvements to this manuscript. We also thank the reviewer for once again elaborating the potential benefit of this work to the field.
